# β-amyloid cytotoxicity is prevented by natural achillolide A

**DOI:** 10.1007/s11418-018-1191-0

**Published:** 2018-03-15

**Authors:** Anat Elmann, Alona Telerman, Rivka Ofir, Yoel Kashman, Orly Lazarov

**Affiliations:** 1 Department of Food Quality and Safety, Agricultural Research Organization, The Volcani Center, POB 15159, 7528809 Rishon LeZion, Israel; 2 The Dead Sea and Arava Science Center, Central Arava Branch, 8682500 Merkaz Sapir, Israel; 3 Raymond and Beverly Sackler Faculty of Exact Sciences, School of Chemistry, Tel Aviv University, Ramat Aviv, 69978 Tel Aviv, Israel; 4 Department of Anatomy and Cell Biology, The University of Illinois at Chicago, Chicago, IL 60612 USA

**Keywords:** Achillolide A, Alzheimer’s disease, Sesquiterpene lactones, Amyloid beta, *Achillea fragrantissima*, Reactive oxygen species

## Abstract

Alzheimer’s disease (AD) is the most prevalent cause of dementia in adults. Current available drugs for AD transiently alleviate some of the symptoms, but do not modify the disease mechanism or cure it. Therefore, new drugs are desperately needed. Key contributors to AD are amyloid beta (Aβ)- and reactive oxygen species (ROS)-induced cytotoxicities. Plant-derived substances have been shown to affect various potential targets in various diseases including AD. Therefore, phytochemicals which can protect neuronal cells against these insults might help in preventing and treating this disease. In the following research, we have isolated the sesquiterpene lactone achillolide A from the plant *Achillea fragrantissima* and, for the first time, characterized its effects on Aβ-treated neuroblastoma cells. Aβ is a peptide derived from the sequential cleavage of amyloid precursor protein, and is part of the pathogenesis of AD. Our current study aimed to determine whether achillolide A can interfere with Aβ-induced processes in Neuro2a cells, and protect them from its toxicity. Our results show that achillolide A decreased Aβ-induced death and enhanced the viability of Neuro2a cells. In addition, achillolide A reduced the accumulation of Aβ-induced ROS and inhibited the phosphorylation of stress-activated protein kinase/c-Jun N-terminal kinase and p44/42 mitogen-activated protein kinase in these cells. We therefore suggest that achillolide A may have therapeutic potential for the treatment of AD.

## Introduction

Alzheimer’s disease (AD) is the most prevalent cause of dementia in adults. Progressive neuronal loss takes place in specific brain areas and causes memory loss, learning difficulty, diminished recall accuracy, impaired problem-solving ability and cognitive deterioration. One of the hallmarks of the disease is the formation of amyloid plaques formed by aggregated β-amyloid (Aβ) peptides. Aβ is a 4-kDa peptide derived from the sequential cleavage of amyloid precursor protein [1] and its oligomeric form is part of the pathogenesis of AD. It is thought to exert its action through different mechanisms, including the induction of reactive oxygen species (ROS) accumulation, microglial activation, and neuronal death [2, 3]. Therefore, phytochemicals that can protect neuronal cells from Aβ toxicity and oxidative stress may assist in coping with AD. Current available drugs for AD transiently alleviate some of the symptoms, but do not modify the disease mechanism or cure it. Therefore, new drugs are desperately needed. Plant extracts and purified phytochemicals have been shown to affect various potential targets in AD such as hyperphosphorylated tau and acetyl cholinesterase activity [4–7].

Sesquiterpene lactones isolated from plants have been shown to possess diverse biological activities and to exhibit several effects, such as immunomodulation, anti-inflammatory, antitumor and antimicrobial activities [8–12]. Sesquiterpene lactones have also been reported to have neuroprotective effects in cultured neurons as well as in vivo, using animal stroke models and cocaine consumption studies. Thus, it is reasonable to assume that these compounds or their active metabolites can cross the blood−brain barrier [8, 13–16]. Based on these activities, sesquiterpene lactones could be promising candidates for the development of drugs for the treatment of neurodegenerative diseases [17–19].

We have previously shown that achillolide A, a sesquiterpene lactone that we isolated from *Achillea fragrantissima* (Forssk) Sch. Bip, downregulated microglial activation [20], prevented hydrogen peroxide (H_2_O_2_)-induced death of astrocytes [21], and protected neuroblastoma cells from glutamate toxicity [22]. Our current study aimed to determine whether achillolide A can interfere with Aβ-induced processes in Neuro2a (N2a) cells and protect them from its toxicity.

## Materials and methods

### Materials

Aβ_25-35_, 2′,7′-dichlorofluorescein diacetate (DCF-DA) and crystal violet were purchased from Sigma Chemical Co. (St Louis, MO, USA). Glutamine, antibiotics (10,000 IU/mL penicillin and 10,000 μg/mL streptomycin), fetal bovine serum (FBS) and Trypin-EDTA were purchased from Biological Industries (Beit Haemek, Israel). Opti-MEM and Dulbecco’s modified Eagle’s medium (DMEM) were purchased from Gibco (Paisley, UK). Dimethyl sulfoxide (DMSO) was obtained from Applichem (Darmstadt, Germany). PathScan total SAPK/JNK sandwich ELISA kit, PathScan phospho-SAPK/JNK (Thr183/Tyr185) sandwich ELISA kit, PathScan total p44/42 MAPK sandwich ELISA kit and the PathScan phospho-p44/42 MAPK (Thr202/Tyr204) sandwich ELISA kit were purchased from Cell Signaling Technology.

### Plant material

The aerial parts of *Achillea fragrantissima* (Forssk) Sch. Bip (*Af*) were collected in the Arava Valley. The plant was authenticated by the botanist Mrs Mimi Ron at The Mount Scopus Botanical Garden in The Hebrew University of Jerusalem. The voucher specimen is kept as part of the Arava Rift Valley Plant Collection under the accession code AVPC0040.

Achillolide A (98% pure) was isolated as previously described from the aerial parts of *Af* [20].

### Preparation of aged Aβ_25-35_

The Aβ_25-35_ peptide was solubilized in sterile double distilled water at a concentration of 2 mM, incubated in a capped vial at 37 °C for 48 h, aliquoted, and stored frozen at −20 °C until use. Fresh dilutions of Aβ were prepared in the growth medium just prior to each experiment, and were used immediately.

### Determination of cytotoxicity

N2a cells were grown and then re-plated onto 96-well plates (5 × 10^3^ cells/well) in 50% Opti-MEM, 43% DMEM (high glucose), 2 mM glutamine, 5% FBS, penicillin at 100 U/mL, and streptomycin at 100 μg/mL. Aβ and/or achillolide A were added 24 h later, and cytotoxicity was determined after 20 h using the lactate dehydrogenase (LDH) activity colorimetric assay (Roche Applied Science, Germany). The absorbance was measured at 492 nm in a Synergy2 Multi-Detection Microplate Reader (BioTek Instruments, Inc., Winooski, VT, USA).

### Determination of cell viability

N2a cells were grown and treated as in the cytotoxicity assay. Cell viability was determined by a modification of the crystal violet assay [23], and the optical density was measured at 540 nm with a 690-nm reference filter in a Synergy2 Multi-Detection Microplate Reader (BioTek Instruments, Inc.).

### Determination of intracellular ROS levels

N2a cells were grown and re-plated as in the cytotoxicity assay, using 1% instead of 5% FBS. After 24 h the cells were treated with 20 µM DCF-DA for 30 min at 37 °C. Following incubation, the cultures were rinsed with phosphate-buffered saline, and fresh medium was added to the cells. The ROS levels before and after treatment with achillolide A and Aβ were determined according to fluorescence (excitation at 485 nm and emission at 520 nm) in a Synergy2 Multi-Detection Microplate Reader (BioTek Instruments, Inc.).

### Determination of total and phospho-SAPK/JNK, and total and phospho-p44/42 MAPK levels

N2a cells were treated with achillolide A and Aβ_25-35_. The cells were lysed after 40 or 30 min for SAPK/JNK or p44/42 MAPK, respectively, in lysis buffer that was part of the ELISA kit. Protein concentrations in cell lysates were determined with Bradford reagent (Bio-Rad, Hercules, CA, USA), and equal amounts of proteins were analyzed by sandwich ELISA kits. The optical density was determined at 450 nm using a multi-detection microplate reader (BioTek Instruments, Inc.).

### Statistical analyses

The results were analyzed by one-way ANOVA followed by Tukey–Kramer multiple comparison tests, using the Graph Pad InStat 3 for Windows (GraphPad Software, San Diego, CA, USA).

## Results and discussion

**Fig. 1:**
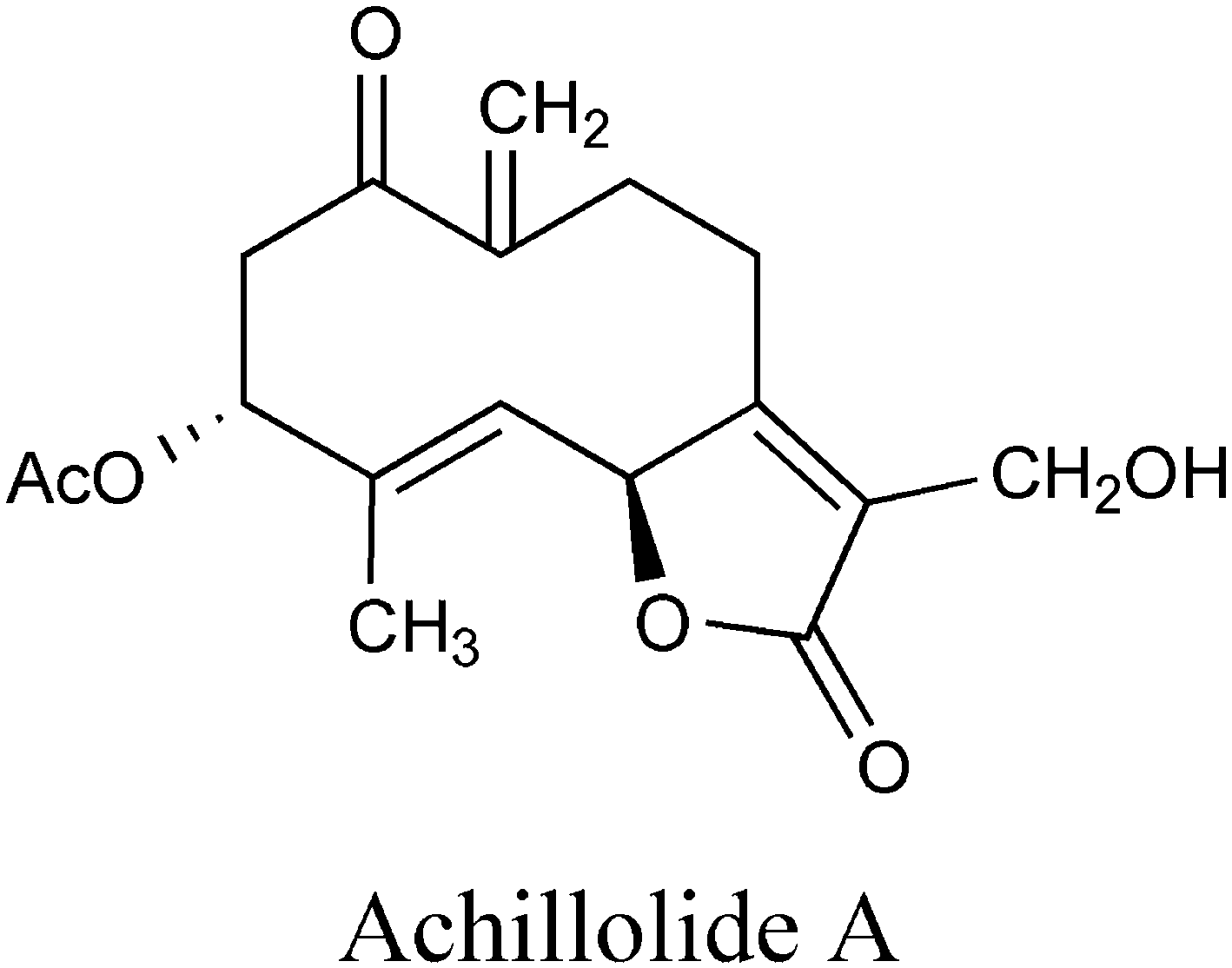
Chemical structure of achillolide A

**Fig. 2:**
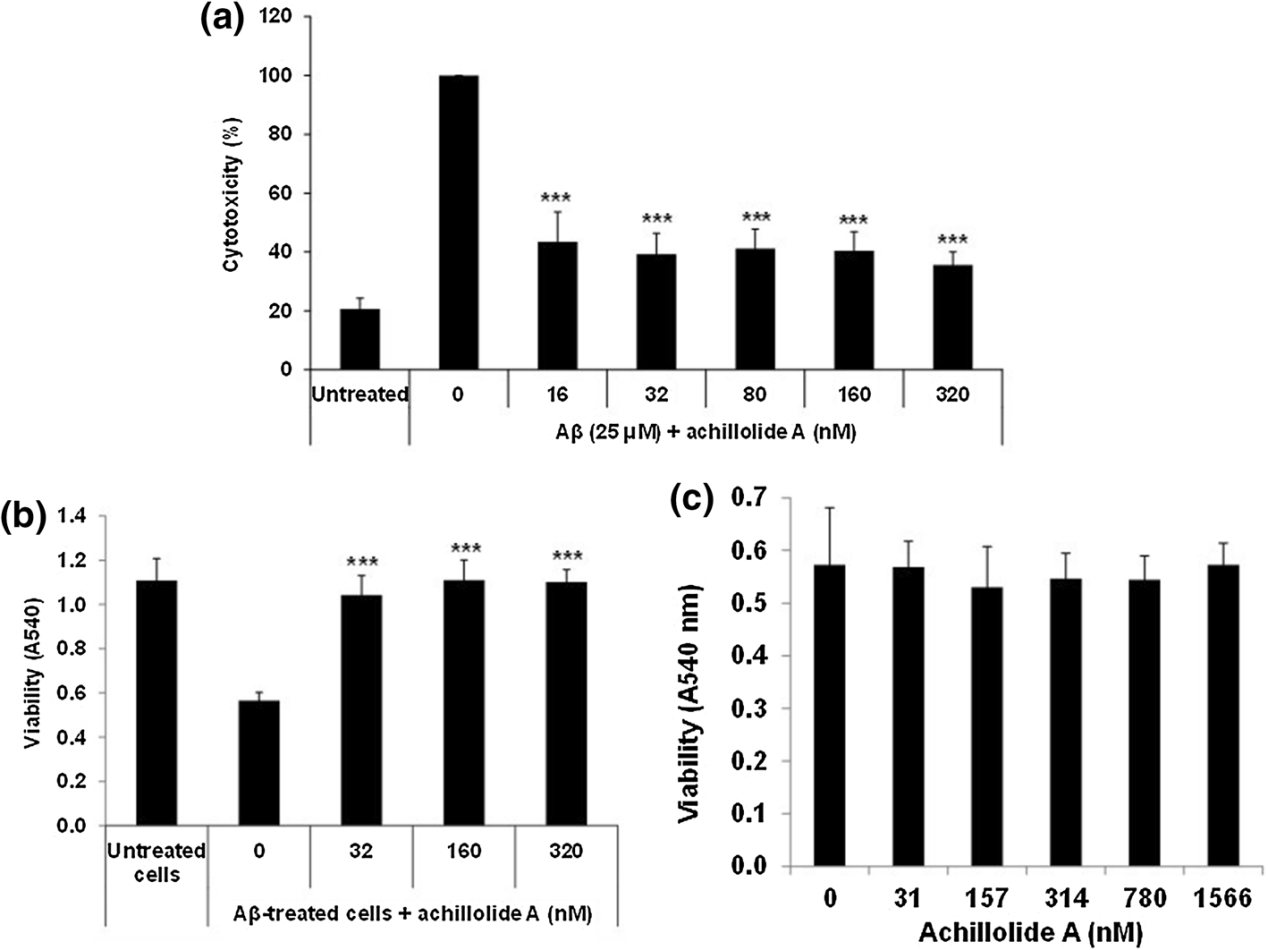
Achillolide A protects N2a cells from Aβ -induced cytotoxicity. Cells were either untreated (‘untreated cells’) or treated with Aβ with or without various concentrations of achillolide A. Twenty hours later cell death was determined by (**a**) the LDH or (**b**, **c**) the crystal violet method. Cytotoxicity (**a**) was significantly reduced in cells treated with Aβ + achillolide A compared to Aβ-treated cells. Likewise, viability (**b**, **c**) was significantly increased in cells treated with Aβ + achillolide A compared to Aβ-treated cells. The results are the mean ± SEM of two experiments (*n* = 16). The maximal LDH release after disruption of the cells by Triton x-100 was A492 = 0.61 ± 0.04 as measured in two experiments (*n* = 5). ***P < 0.001

Achillolide A (Fig. 1) was previously shown by us to inhibit microglial activation, protect astrocytes from oxidative stress, and protect neuroblastoma cells from glutamate toxicity [22, 24]. Aβ_25-35_ is a neurotoxic peptide commonly used in cellular models of AD [25, 26]. We have previously shown that exposure of N2a neuroblastoma cells to Aβ_25-35_ resulted in their death 20 h later [22]. To examine the effect of achillolide A on the toxicity of Aβ, these cells were treated with 25 μM of the Aβ_25-35_ peptide together with different concentrations of achillolide A. Viability and cytotoxicity were determined 20 h later. Our results show that achillolide A reduced Aβ_25-35_-induced cell death by 71% at a concentration of 16 nM (Fig. 2a), as observed by the LDH method, and completely rescued viability, as observed by the crystal violet method (Fig. 2b). It should be noted that achillolide A by itself is not cytotoxic to N2a cells that were exposed to different concentrations (up to 1566 nM) of this molecule, as determined using the crystal violet assay (Fig. 2c).

**Fig. 3:**
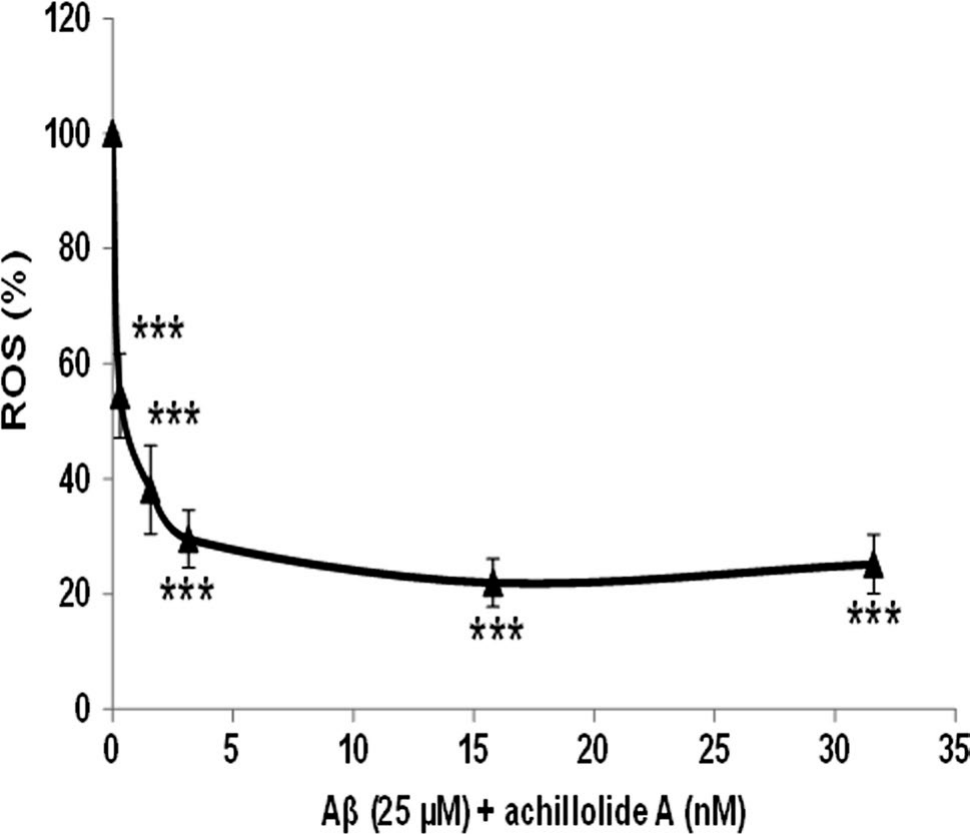
Achillolide A attenuates ROS levels induced by Aβ in N2a cells. Levels of ROS were significantly reduced in N2a cells treated with Aβ + achillolide A compared to Aβ-treated cells. The results represent the mean ± SEM of three experiments (*n* = 24). *p < 0.05, ***p < 0.001

One mechanism by which Aβ may exert cell toxicity is the generation of ROS, leading to neuronal death [3]. Treatment of N2a cells with Aβ_25-35_ for 20 h results in a two-fold increase in intracellular ROS levels, as we have previously shown [22]. We therefore tested whether achillolide A could inhibit the elevated production of ROS following treatment with Aβ_25-35_ and, as a result, protect the cells from Aβ_25-35_-induced cytotoxicity. To test this possibility, cells were treated with various concentrations of achillolide A at the time of Aβ_25-35_ application, and ROS formation was determined 20 h later. Our results show that treatment with achillolide A inhibits 78% of the intracellular levels of Aβ_25-35_-induced ROS. We observed that 16 nM achillolide A is the lowest effective dose at both attenuating neuronal cell death and reducing the level of ROS production following Aβ_25-35_ treatment (Figs. 2, 3).

The antioxidant characteristics of achillolide A demonstrated in this study in Aβ-treated N2a neuroblastoma cells support our previous observations, showing similar effects in glutamate-treated neuroblastoma N2a cells [22], H_2_O_2_-treated astrocytes and LPS-activated microglial cells [20, 24]. Although part of Aβ toxicity is mediated by glutamate [27], the mechanism underlying Aβ cytotoxicity is complex and involves many downstream targets (for review see [28]). As neuronal vulnerability in AD originates in the hippocampal formation, future experiments should examine the effect of achillolide A on these neuronal populations in vitro and in vivo.

Enhanced activation of SAPK/JNK and p44/42 MAPK was observed following treatment of cells with Aβ [29–31], as well as in brains of AD patients [32–34]. Since MAPK signaling was shown to be modulated by sesquiterpene lactones [35–37], we determined the effect of achillolide A on the phosphorylation of SAPK/JNK and p44/42 MAPK by Aβ_25-35_.

**Fig. 4:**
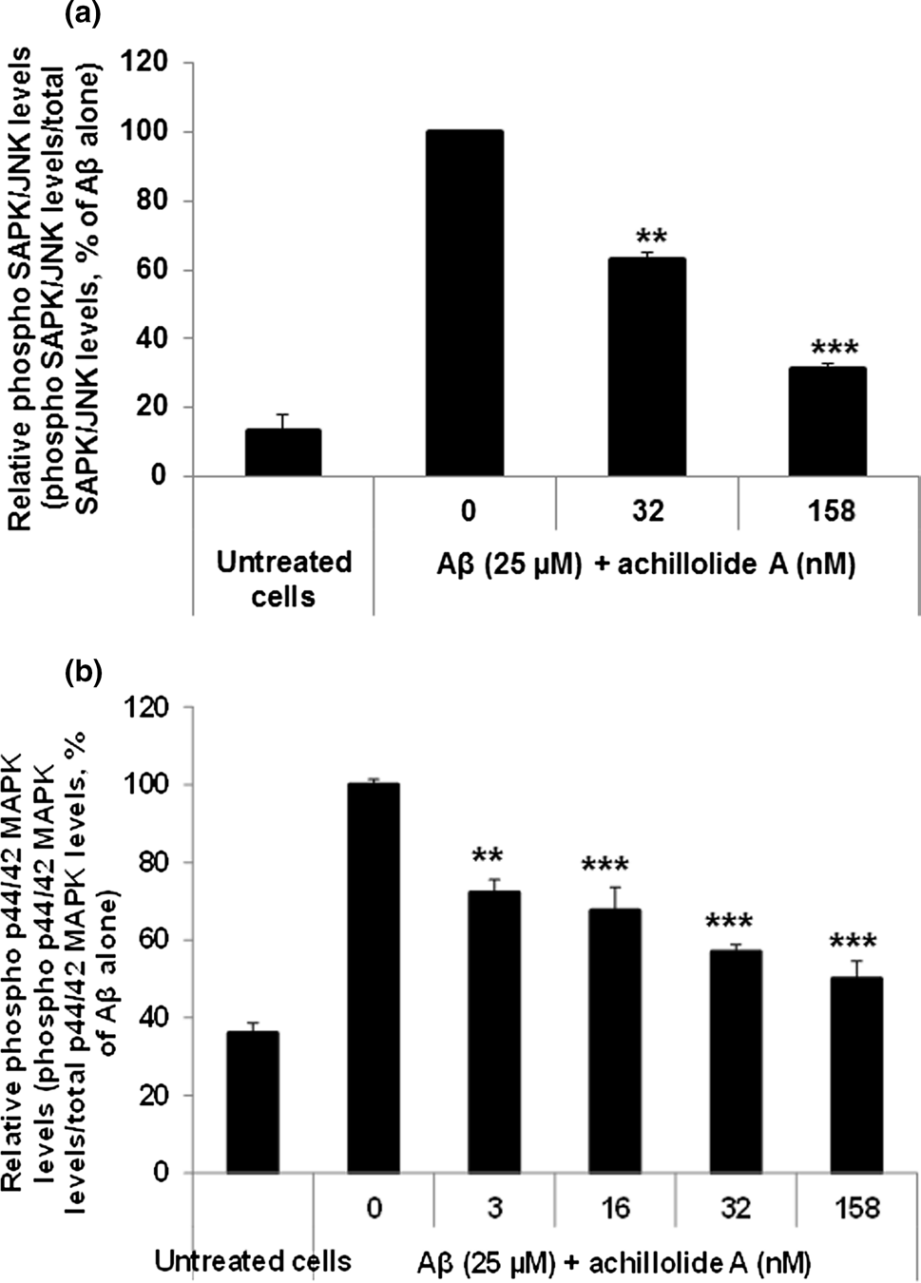
Achillolide A attenuates the phosphorylation of p44/42 MAPK and SAPK/JNK induced by Aβ in N2a cells. Cells were either untreated or treated with Aβ only (25 μM) or Aβ + achillolide A for 30 min (p44/42 MAPK) or 40 min (SAPK/JNK). The levels of phosphorylated and total SAPK/JNK (**a**) and p44/42 MAPK (**b**) in cell extracts were determined by corresponding ELISA kits. The levels of the phosphorylated proteins were normalized to the levels of the total amount of the related proteins, and are presented as the mean ± SEM of two experiments (*n* = 4) for SAPK/JNK, and three experiments (*n* = 6) for p44/42 MAPK. The levels of the phosphorylated proteins were significantly lower in cells treated with both Aβ + achillolide A compared to cells treated with Aβ only. **p < 0.01, ***p < 0.001

Figure 4 shows that Aβ_25-35_ increased the phosphorylation of SAPK/JNK by 7.5-fold in N2a cells, and achillolide A (at 158 nM) inhibited 79% of Aβ_25-35_-induced phosphorylation (Fig. 4a). At the same concentration, achillolide A also inhibited 78% of the Aβ_25-35_-induced phosphorylation of p44/42 MAPK (Fig. 4b), without affecting the total amount of these proteins in the cells (data not shown). These results suggest that inhibition of the Aβ-induced phosphorylation of SAPK/JNK and p44/42 MAPK is part of the mechanism by which achillolide A protects neurons against Aβ_25-35_ toxicity.

## Conclusions

In this study, we have shown for the first time that achillolide A, a natural sesquiterpene lactone we isolated from *A. fragrantissima*, can protect N2a cells from Aβ_25-35_-induced cell death. In addition, achillolide A reduced the accumulation of Aβ-induced ROS in N2a cells and inhibited the phosphorylation of SAPK/JNK and p44/42 MAPK in these cells. Based on our results in astrocytes, microglial cells and N2a neuroblastoma cells, it is proposed that achillolide A has neuroprotective therapeutic characteristics. Further studies are warranted in order to substantiate the therapeutic potential of achillolide A for the treatment of AD.
